# Astrocytogenesis: where, when, and how

**DOI:** 10.12688/f1000research.22405.1

**Published:** 2020-04-02

**Authors:** Ekin Su Akdemir, Anna Yu-Szu Huang, Benjamin Deneen

**Affiliations:** 1 Center for Cell and Gene Therapy, Baylor College of Medicine, Houston, TX, 77030, USA; 2 Program in Developmental Biology, Baylor College of Medicine, Houston, TX, 77030, USA; 3 Department of Neuroscience, Baylor College of Medicine, Houston, TX, 77030, USA; 4 Department of Neurosurgery, Baylor College of Medicine, Houston, TX, 77030, USA

**Keywords:** Glia, Astrocyte, Gliogenesis, Neurodevelopment

## Abstract

Astrocytes are the most abundant cell type in the central nervous system and have diverse functions in blood–brain barrier maintenance, neural circuitry formation and function, and metabolic regulation. To better understand the diverse roles of astrocytes, we will summarize what is known about astrocyte development and the challenges limiting our understanding of this process. We will also discuss new approaches and technologies advancing the field.

## Introduction

The central nervous system (CNS) is composed of multiple highly specialized cell types, including neurons, astrocytes, oligodendrocytes, microglia, and endothelial cells, each with different functions that are highly adapted to local circuitries ^1– 3^. Astrocytes are the most abundant glial cells in the CNS, representing between 20 and 40% of total cells in the brain ^4, 5^. Astrocytes were first described by Rudolf Virchow as a homogenous connective tissue supporting nervous system elements. Later, Ramon y Cajal and others investigated the cellular structure of the brain and observed that different brain regions contain morphologically distinct neuronal and glial cell types.

Over the past century, astrocytes have emerged as a bridge to the outside world for neurons with diverse physiological roles in neural development, neural circuit function, neurotransmission, blood–brain barrier formation, and metabolic support. Astrocytes form the neurovascular unit between neurons and endothelial cells, and their end-feet structure maintains brain homeostasis by regulating water, amino acid, and neurotransmitter uptake. Astrocytes also have the ability to monitor the ongoing local activity of synaptic circuits with their elaborate processes ensheathing synapses and forming tripartite synapses. Astrocytes express neurotransmitter receptors that sense synaptic activity and respond to it by elevating astrocytic Ca ^2+^ and secreting neuroactive molecules back to synapses. Despite these known roles, how astrocytes develop and mature to form functional neurovascular circuits to carry out these diverse functions remains relatively unknown ^1– 4^.

Previous studies have shed light on how neurons and oligodendrocytes develop in a stepwise fashion, where they specify, undergo terminal differentiation, and enter the postmitotic stage. Since the initial observation by Ramon y Cajal, hundreds of types of neurons have been identified and functionally characterized, while astrocytes are still broadly classified as either protoplasmic or fibrous. The lack of markers and tools to access the precursor and intermediate stages of astrocyte development has hindered characterization of their development and heterogeneity. Further complicating their characterization, astrocytes are more plastic than neurons and proliferate after specification. However, with their critical and diverse functions that actively regulate neuronal function, it is essential to understand where, when, and how astrocytes are generated during development.

## Stages of astrocyte development

Neural stem cells (NSCs), or radial glia, generate astrocytes through complex intrinsic and extrinsic cellular processes. This sequential series of events results in mature astrocytes that actively participate in CNS physiology. Conserved mechanisms regulate gliogenesis in the spinal cord and brain, although the process begins early, at embryonic day 11.5 (E11.5), in the spinal cord and later, at E18, in the brain. Whereas spinal cord astrocytes are derived from the ventricular zone (VZ), forebrain astrocytes are from the ventricular–subventricular zone (V-SVZ) ^6^. Below, we discuss recent progress toward understanding astrocyte development and maturation, beginning with patterning and specification, proliferation, and maturation.

## Astrocyte specification and developmental patterning

**Figure 1.**
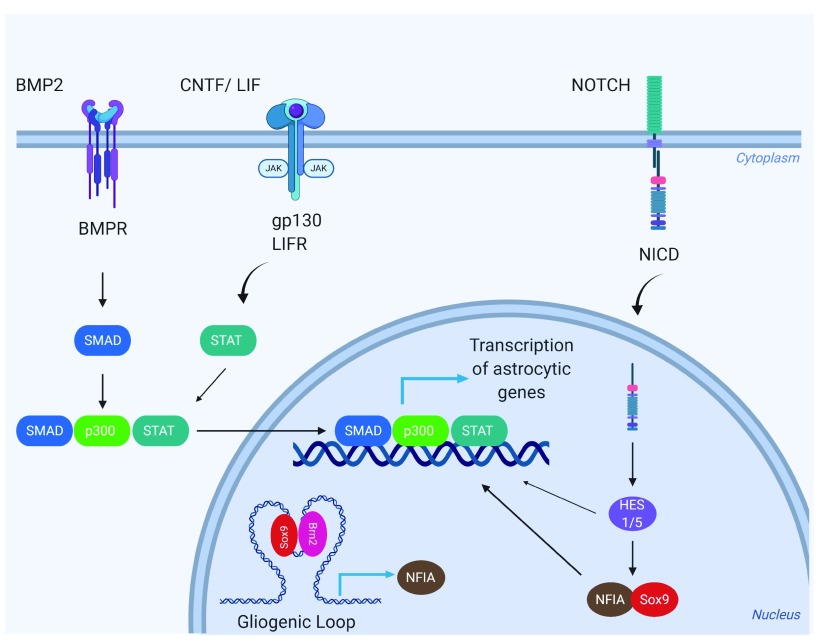
Molecular signaling pathways involved in astrocytogenesis. Neural stem cells (NSCs) express astrocytic genes in response to several signaling molecules, including bone morphogenic protein (BMP) families, the leukemia inhibitory factor/ciliary neurotrophic factor (LIF/CNTF), and Notch pathway. BMPs signal primarily through SMAD, whereas LIF/CNTF activates the JAK/STAT pathways. The active SMAD–STAT complex bridged by p300 goes directly into the nucleus, binds to the promoter, and activates astrocytic genes such as *GFAP* and *S100*. Another important pathway that regulates astrocytogenesis is the Notch pathway. Notch ligands will activate Notch receptors and activate the expression of Hes genes, *Hes1/5*. Hes1, Hes5, and activated forms of Notch receptors induce the expression of astrocytic genes and glial promoting transcription factor nuclear factor-I A (NFIA). In early development, a pre-formed gliogenic loop serves to facilitate the association between sex-determining region Y-box 9 (Sox9) and brain-specific homeobox/POU domain protein 2 (Brn2), which also drives expression of NFIA. NFIA is both necessary and sufficient for the induction of astrocytic genes.

During CNS development, neurons are specified from NSCs before glial cells, and radial glia serve as a scaffold for this process. Signaling pathways and dynamic transcription factor expression control these cell fate decisions. Previous studies have focused on the spinal cord VZ, where the timing of the gliogenic switch in NSCs is clearly defined. In the VZ, neurogenesis ceases at E11.5 and gliogenesis commences at E12.5, and transcription factors sex-determining region Y-box 9 (Sox9) and nuclear factor-I A (NFIA) play critical roles during this developmental interval. Sox9 and brain-specific homeobox/POU domain protein 2 (Brn2) regulate NFIA induction and glial specification ^7^. NFIA is both necessary and sufficient for embryonic gliogenesis ^8^, and the association between Sox9 and NFIA regulates genes essential for astrocyte migration and maturation ^9^ ( Figure 1). In neocortical development, zinc finger- and BTB domain-containing protein 20 (Zbtb20) was shown to promote astrocyte specification while suppressing the production of oligodendrocyte precursors (OPCs), and knockdown of either NFIA or Sox9 suppresses Zbtb20 activity ^10^.

Notch signaling is another important regulator of cell differentiation. In early development, Notch activation maintains the NSC pool in addition to inducing NFIA. NFIA maintains the continued inhibition of neurogenesis through induction of the Notch effector Hes5, further clarifying the role of Notch signaling in early gliogenesis ^8^. NFIA associates with astrocytic gene promoters such as *GFAP* to disrupt their methylation through association with DNA methylating dissociation of the DNA methylating enzyme 1 (Dnmt1) ^11, 12^. Loss of function of Notch effectors such as Hes5 leads to loss of glia, and gain of function produces more glia; however, Notch activity alone is insufficient to drive precocious astrocytic differentiation; thus, Notch signaling is permissive and not instructive for gliogenesis ^13^ ( Figure 1).

During early development, the embryonic spinal cord is patterned throughout the dorsoventral axis by a combination of morphogens—sonic hedgehog (Shh), BMPs, and Wnts—which regulate the combinatorial expression of homeodomain transcription factors ^14^. These patterning principles govern what type of progenitor cells will be generated from.

The first evidence of patterning during astrocyte development emerged in 2005, when Muroyama *et al*. showed that the basic helix-loop-helix (bHLH) transcription factor Scl/tal1 (stem cell leukemia, Tal) promotes astrogenesis via a cross-antagonistic relationship with the oligodendrocyte transcription factor Olig2 ^15^. At the p2 and pMN domain boundary, the interaction between Scl and Olig2 allowed p2 domain to generate astrocyte precursors while the pMN domain produced oligodendrocyte precursors ^15^.

When expressed in different VZ domains, the transcription factors Pax6 and Nkx6.1 yield three distinct astrocyte subtypes (VA1, VA2, and VA3, dorsal to ventral) that arise from the p1, p2, and p3 progenitor domains, respectively ^16^. These populations are further demarcated by differential expression of Reelin and Slit. Tsai *et al*. used lineage tracing with Cre recombinases to specifically target these domains and demonstrated that astrocytes arise from different segmental domains throughout the brain and spinal cord ^17^. When specific astrocyte populations were ablated with a Cre-dependent diphtheria toxin A (DTA) approach, the authors observed that neighboring astrocytes from other progenitor domains were unable to migrate, suggesting that there are region-specific neuron–astrocyte interactions following developmental patters. This domain-specific identity is also observed in forebrain astrocytes, which showed dorsal-ventral restriction depending on the developing region they derived from, as a result of strictly radial migration ^17^. Overall, these studies illuminate the transcriptional code determining astrocytes’ regional identity and suggest that patterning also may lead to functional diversity.

Before terminal differentiation, astrocyte precursors migrate from VZ and SVZ along the radial glial processes. Newborn astrocytes continue to divide locally after migration. Initial studies identified these targeted precursor astrocytes with Glast ^18^, a glutamate aspartate transporter whose induction coincides with the gliogenic switch. A recent study identified new glial markers for the intermediate stages of astrocyte development using a Glast reporter mouse, including *Asef*, *Gpr37l1*, *Mfge8*, and *Tom1l1* ^19^. Asef is a guanine exchange factor that has a role in cell migration, and functional studies *in vivo* have shown that Asef is required for AQP4 expression on the end-feet of spinal cord astrocytes ^19^.

Our understanding of the stepwise specification and migration of astrocytes is improving with newly developed tools for cell lineage mapping, spatial and temporal profiling, and functional studies. However, several important questions remain. For instance, which other factors trigger the gliogenic switch in NSCs is an ongoing area of investigation. Furthermore, owing to the lack of markers for astrocyte precursors, it has been difficult to dissect the signaling mechanisms responsible for astrocyte specification and migration. Whether we can use the recently identified and functionally characterized intermediate markers to address these remains an open question.

## Astrocyte differentiation

The differentiation trajectory of astrocytes in the postnatal mouse brain remains somewhat controversial ^10, 20, 21^. In one study, Nagao *et al*. identified astrocyte-lineage restricted progenitors using astrocyte lineage-specific marker Zbtb20 along with markers that label both astrocyte and oligodendrocyte precursors, Sox9 and Olig2 ^10^. Using immune co-staining, the group identified the presence of Zbtb20 ^+^/Sox9 ^+^/Olig2 ^+^ triple-positive cells in the mouse postnatal day 3 (P3) neocortex and demonstrated that Zbtb20 ^+^/Olig2 ^+^ cells were devoid of Sox10, a marker of OPCs ^10^. However, in other studies, several groups have identified progenitor cells that exhibited bipotent signatures, expressing cell markers of both the astrocytic (Aldh1l1, AldoC, Aqp4, and Slc1a3) and oligodendrocytic (Pdgfra, Olig1, and Olig2) lineages by microarray, immunofluorescent co-staining, and single-cell sequencing ^20, 21^. This inconsistency may stem from the current use of a single marker (for example, Aldh1l1 only or GFAP only) to identify astrocytes. Similar to the hematopoietic system, specific lineages may be better characterized by combinatorial codes of multiple cell markers. Recent advances in single-cell sequencing technology should enable us to discover and validate combinations of cell markers that better demarcate the intermediate stages of the astrocyte lineage.

Astrocytes express canonical markers before initiating terminal differentiation. One of the markers expressed at a later stage is glial fibrillary acidic protein (GFAP), which provides structural stability and motility to astrocytes. Several groups generated *GFAP-Cre* mouse lines to target astrocytes, but these lines also target neural progenitor cells ^11– 13, 18, 22^. Although GFAP is commonly used as a marker, it is weakly expressed in protoplasmic astrocytes in rodents. Other currently used astrocytic markers include calcium-binding protein S100 ^23^, glutamate transporter GLAST ^24^, aldolase C ^25^, CD44 ^26^, glutamine synthase (GS), and fatty acid-binding protein FABP7. Aquaporin 4 (AQP4) and connexins 30 (Cx30) have been used as astrocyte end-feet markers ^27, 28^. These markers are less specific than GFAP, as all of them also can be expressed in neurons, oligodendrocytes, or ependymal cells ^14^. Glutamate transporter, EAAT2, also known as GLT-1, is expressed in astrocytes and neurons, although 80% of total EAAT2 protein is expressed in astrocytes in the hippocampus ^29^. These markers can be used in a combinatorial approach. For example, Miller *et al*. used a specific promoter region of *GLT-1* to generate a reporter mouse that targets gray matter astrocytes in the cerebral cortex, which interestingly are absent in the hippocampus ^30^. The authors further analyzed the transcriptome of this astrocytic population and determined its unique molecular profile, identifying a pathway-specific to it with the expression of the genes *norrin* and *LRG6*, which have roles in dendritic spine maintenance in this population ^30^. (Also, see the “Molecular maturation” section).

Recent genetic profiling studies identified aldehyde dehydrogenase family 1, member L1 (ALDH1L1), a metabolic enzyme, as the most homogenously expressed astrocyte marker throughout the brain. Aldh1L1 was reportedly used to stain cortical astrocytes, whereas hippocampal astrocytes are widely stained with GFAP ^31^. Morel *et al*. also used a combinatorial approach with the *Aldh1L1-GFP* transgene reporter mouse with the EAAT2-tdtomato mouse line and identified a tdT ^−^eGFP ^+^ astrocyte population that is selectively localized at layers I and II in the cortex ^31^. Furthermore, this population of cells showed increased resting membrane potential and resistance and reduced potassium channel Kir4.1 expression ^31^. The recently developed *Aldh1L1-CreER* transgenic mice should allow astrocyte-specific manipulations ^32^.

Transcription factors NFIA and SOX9 are also non–stage-specific astrocytes markers, although NFIA also is expressed in oligodendrocyte precursor cells and some neurons. On the other hand, Sox9 is not expressed in neurons and recently was identified as an astrocyte-specific marker in the adult brain. Henceforth, Sox9 may be an important tool to access astrocytes in the adult brain ^32– 34^.

GFAP expression is used as an indicator of astrocyte maturation and has given significant insight into the mechanisms regulating astrocyte differentiation. Previously, it was used to identify the JAK-STAT pathway and BMPs and Notch signaling as central players controlling astrocyte differentiation from precursor cells ^35, 36^. Recently, a large-scale interchromosomal interaction study identified *Brahma-related gene 1* (BRG1), an ATP-dependent chromatin remodeling factor, as clustering with the *GFAP* gene and regulating GFAP expression ^37^.

In addition to these general astrocyte markers, astrocyte markers for anatomically distinct populations have been identified in recent studies. Molofsky *et al*. used the *Aldhl1L1-GFP* transgene reporter mouse to identify distinct molecular differences between dorsal and ventral astrocytes and identified Sema3a, an axon guidance protein, as being highly expressed in ventral astrocytes ^38^. Ventral region motor neurons α–MN failed to maintain axon initial segment orientation following loss of astrocytic Sema3a, affecting their survival and function. That study was the first to show how positional cues by diverse astrocytes maintain specific circuitry ^38^. Inwardly rectifying potassium channel, Kir4.1, also was shown to be enriched in astrocytes of the spinal cord ventral horn, which support the survival and function of motor neurons ^39^. Another study compared adult striatal and hippocampal astrocytes and identified μ-crystallin Crym as a striatal astrocyte-specific marker ^2^. Although the functional significance of this protein is unknown, it is the first marker that defines a region-specific astrocyte population in the brain. These studies indicate that the molecular and anatomical properties of astrocyte subpopulations may yield insight into their function.

Although our knowledge of astrocyte biology continues to expand, how mature astrocytes are formed and differentiate to carry out their diverse roles remains unclear. Historically, GFAP has been used to understand how external inputs affect astrocyte maturation, even though it is not the most comprehensive astrocyte marker in rodents. In the future, identifying region-specific astrocyte markers may allow us to study specific circuits. In addition, we have not yet identified a developmental endpoint for astrocytes. In the CNS, terminally differentiated neurons and oligodendrocytes are postmitotic while GFAP-expressing astrocytes retain their ability to proliferate (discussed in further detail in the next section).

## Astrocyte proliferation

Neurogenesis is complete in most regions of the brain at birth, and the same number of neurons is maintained throughout life ^40^. On the other hand, concurrent with brain growth during this period, the number of glial cells increases six- to eight-fold during the first three weeks of postnatal development ^41^. Given the diverse functions of astrocytes, which facilitate maturation of the neuronal network during this critical developmental period ^42^, it is important to understand where, when, and how astrocytes proliferate to reach their final population size.

Over the past few decades, it has become clear that cortical astrocytes are generated from four main sources in successive yet overlapping chronological order ^43– 45^. Each stage of development takes place in a specific anatomical region; astrocyte differentiation first occurs from radial glia in the VZ of late embryonic-perinatal brain (1), followed by astrocytes in the cortex of postnatal brain (2), progenitor cells in the SVZ of postnatal and adult brain (3), and NG2 cells in the cortex of postnatal or adult brain or both (4). However, as discussed above, astrocytes in the developing spinal cord are generated mainly from radial glia and astrocyte progenitors derived from radial glia at an earlier developmental stage. Below, we will focus on developmental astrocytogenesis from the VZ and local proliferation in the cortex, which are major sources of astrocytogenesis and together contribute to about 80% of cortical astrocytes ^45^. Meanwhile, we will briefly describe the process of astrocyte proliferation in the spinal cord. For SVZ and NG2 cell-derived astrocytes in the cortex, please see reviews ^43– 45^.

### Astrocyte generation in the ventricular zone

Radial glia are bipolar cells with their soma residing in the VZ of the embryonic CNS. Radial glia extend and anchor one branch to the ventricular wall and the other to either the pial surface or blood vessels ^46– 50^. During the late embryonic stage, radial glia are the major source of astrocytogenesis, and their bipolar structure provides a scaffold for the migration of newborn astrocyte precursor cells ^50^.

**Figure 2.**
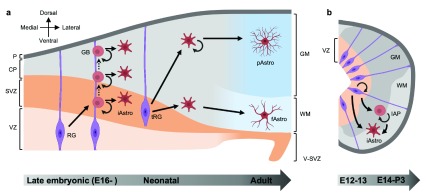
Astrocytogenesis from the ventricular/subventricular zones and outer cortical layers in the mouse developing central nervous system (CNS). ( **a**) In the developing cortex, radial glia (RG) first give rise to glioblasts (GBs) during the late embryonic to perinatal period. Glioblasts undergo several rounds of division while migrating out along radial glia, resulting in clusters of astrocytes in the developing cortex. At the terminal stage of radial glial differentiation, radial glia detach from the ventricular zone and form unipolar transitional radial glia (tRG), which give rise to protoplasmic and fibrous astrocytes in the gray matter and white matter of the cortex, respectively. During the early postnatal period, differentiated astrocytes in the outer cortical layer undergo symmetric division and generate daughter astrocytes that exhibit astrocytic morphology and functions. ( **b**) In the developing spinal cord, radial glia first proliferate during embryonic day 12 (E12) to 13, giving rise to radial glial pool which differentiates into astrocytes between E14 and postnatal day 3 (P3). Alternatively, radial glial cells differentiate into intermediate astrocyte precursors (IAPs), which proliferate during E14 to P3 and undergo terminal differentiation, ultimately giving rise to astrocytes. The progression from embryonic stage to adult is shown from left to right below each panel. Straight arrows indicate differentiation or maturation from one cell type to another. Circular arrows indicate proliferation. Dashed arrows indicate migration. CP, cortical plate; fAstro, fibrous astrocyte; GM, gray matter; iAstro, immature astrocyte; P, pia mater; pAstro, protoplasmic astrocyte; SVZ, subventricular zone; VZ, ventricular zone; WM, white matter.

Astrocytogenesis from radial glia in the developing cortex occurs in two waves. In the first wave, glia progenitors or glioblasts are derived from the asymmetrical division of radial glia. Glioblasts are proliferative glia progenitors found between the late embryonic (E16–E18) and perinatal stage in the mouse cortex ^50, 51^. Upon generation, these glia progenitors migrate radially from the VZ/SVZ and undergo several rounds of proliferation on their way out, giving rise to multiple clusters of astrocytes in the same cortical column of the postnatal cortex ^52– 54^. The second wave, which occurs at the terminal stage of differentiation, results from the direct transformation of radial glia. Between the late embryonic and early perinatal stage, radial glia detach their anchorage from VZ and lift their soma toward the pial surface, resulting in unipolar transitional radial glia (tRG). These tRG undergo terminal differentiation to give rise to protoplasmic and fibrous astrocytes in the gray matter and white matter of cortex, respectively ( Figure 2a). Using retroviral-mediated lineage tracing, organotypic slice culture, and confocal time-lapse imaging on rat brain slices at E16, Noctor *et al*. demonstrated direct evidence that individual radial glial cells transform into astrocytes at the terminal stage of differentiation ^49^. The presence of tRG-derived astrocytes has been seen across several different species, including monkey, ferret, human, and rodents, suggesting an evolutionarily conserved mechanism that controls terminal differentiation of radial glia into astrocytes ^46– 50^.

Parallel to findings in the developing cortex, astrocytes in the spinal cord are also derived from radial glia, albeit earlier, between E14 and P3 ^17, 55, 56^. Using *Aldh1L1-GFP* labeling, Tien *et al*. found two types of proliferative astrocyte precursors that exhibit distinct morphology ^56^. Radial glia in the VZ were the first precursors identified, and the second were astrocyte progenitors called intermediate astrocyte precursors (IAP) ^56^. These IAPs are generated first from radial glia before migrating out of the VZ into the mantle zone, where they undergo several rounds of division before terminally differentiating into spinal cord astrocytes ( Figure 2b).

Overall, these studies demonstrate that a conserved mechanism exists between the embryonic cortex and spinal cord, wherein radial glial cells first give rise to lineage-restricted glia progenitors, which then undergo multiple rounds of division to give rise to astrocytes in the postnatal CNS.

### Astrocyte proliferation in the outer cortical layer

Early lineage-tracing studies with VZ/SVZ progenitors identified clusters of astrocytes in the postnatal cortex, indicating local proliferation of cells during or after migration or both ^51, 52, 57^. Although the field long hypothesized that these clusters derived from glioblasts ^50, 51, 56^, one study found that local proliferation of terminally differentiated astrocytes in the outer cortical layer (I–IV) of the early mouse postnatal brain (P0–P2) may have contributed to some of these clusters ^58^ ( Figure 2a). These parental cells exhibited astrocyte characteristics, including morphology, gap junction connectivity, and expression of astrocyte markers; however, they underwent symmetrical division to give rise to two functional daughter astrocytes. This symmetrical division was at its highest rate before P6 and decreases over time, resulting in the generation of 50% of total astrocytes at P28 ^58^. Consistently, Moroni *et al*. identified, in the rat postnatal cortex, astrocytes that expressed differentiated astrocyte markers S100b or Aldh1L1 that co-expressed proliferation marker Ki67 ^59^. The proliferation rate of these cells increased from P1 to P10 and decreased thereafter ^59^. The consistent timeline between mouse and rat suggests a conserved mechanism regulating the proliferative potential of these differentiated astrocytes. Conversely, in the adult SVZ, lineage tracing demonstrated that adult SVZ-derived astrocytes are mostly postmitotic with no signs of local proliferation ^60^. These studies suggested that astrocytes derived from different sources may have different proliferative potential once differentiated. Henceforth, it will be important to delineate the mechanisms that endow postnatal astrocytes with their unique proliferative potential.

Despite the above discoveries, several unanswered questions remain in the field. For example, since not all differentiated astrocytes proliferate ^58^, it will be important to uncover which mechanisms drive the transition between dividing to non-dividing astrocytes. Interestingly, Ge *et al*. demonstrated that dividing astrocytes exhibit slight changes in their membrane properties compared with surrounding non-dividing astrocytes, suggesting that dividing astrocytes retain or regain proliferative activity, which could result from local environmental cues or cell-intrinsic molecular mechanisms or both ^58^. Indeed, several previous studies identified clusters of astrocytes residing around specific structures of the brain, including blood vessels, the pial surface, and corpus callosum, or specific layers of the cortex ^58, 61^, indicating the presence of local environmental cues. As of today, we still know very little about the cell-intrinsic mechanisms underlying the local proliferation of differentiated astrocytes. Recent studies identified that YAP (yes-associated protein), a transcription co-factor of the Hippo signaling pathway, is required for the proliferation of astrocytes in postnatal neocortex via cooperation with the BMP-SMAD signaling pathway ^62^. However, more detailed study on the *in vivo* brain is needed. With new single-cell sequencing techniques, we should be able to delineate the transcriptomes of these dividing astrocytes and thus identify signaling pathways and associated environmental cues that alter gene expression to confer these dividing astrocytes with proliferative activity.

Studies during the embryonic stage have delineated gliogenesis during development, but our understanding of postnatal astrocytogenesis is still in the early stage. Answering the above questions should shed light on the mechanisms controlling postnatal astrocyte proliferation, which may be further applied in different contexts, including reactive gliosis and glioma.

## Astrocyte maturation

For more than 100 years, astrocytes have been divided into two main subtypes—protoplasmic or fibrous—on the basis of differences in their cellular morphologies and location. During the late phase of astrocyte proliferation, astrocytes undergo morphological and molecular maturation to develop their characteristic “spongiform” morphology and tiny distal processes called perisynaptic astrocytic processes (PAPs). Protoplasmic astrocytes of the gray matter have PAPs with several stem branches, giving rise to fine branches that ensheathe neural synapses and form direct contact with blood vessels. Fibrous astrocytes of white matter have an elongated morphology and are in contact with myelinated axonal tracts and nodes of Ranvier ^63^. Concurrent with morphological maturation, both astrocyte types begin to express functional proteins, including channels and receptors in their membrane, and secrete synaptogenic factors. Below, we summarize the current understanding of morphological and molecular astrocyte maturation and discuss their functional implications.

### Morphological maturation

**Figure 3.**
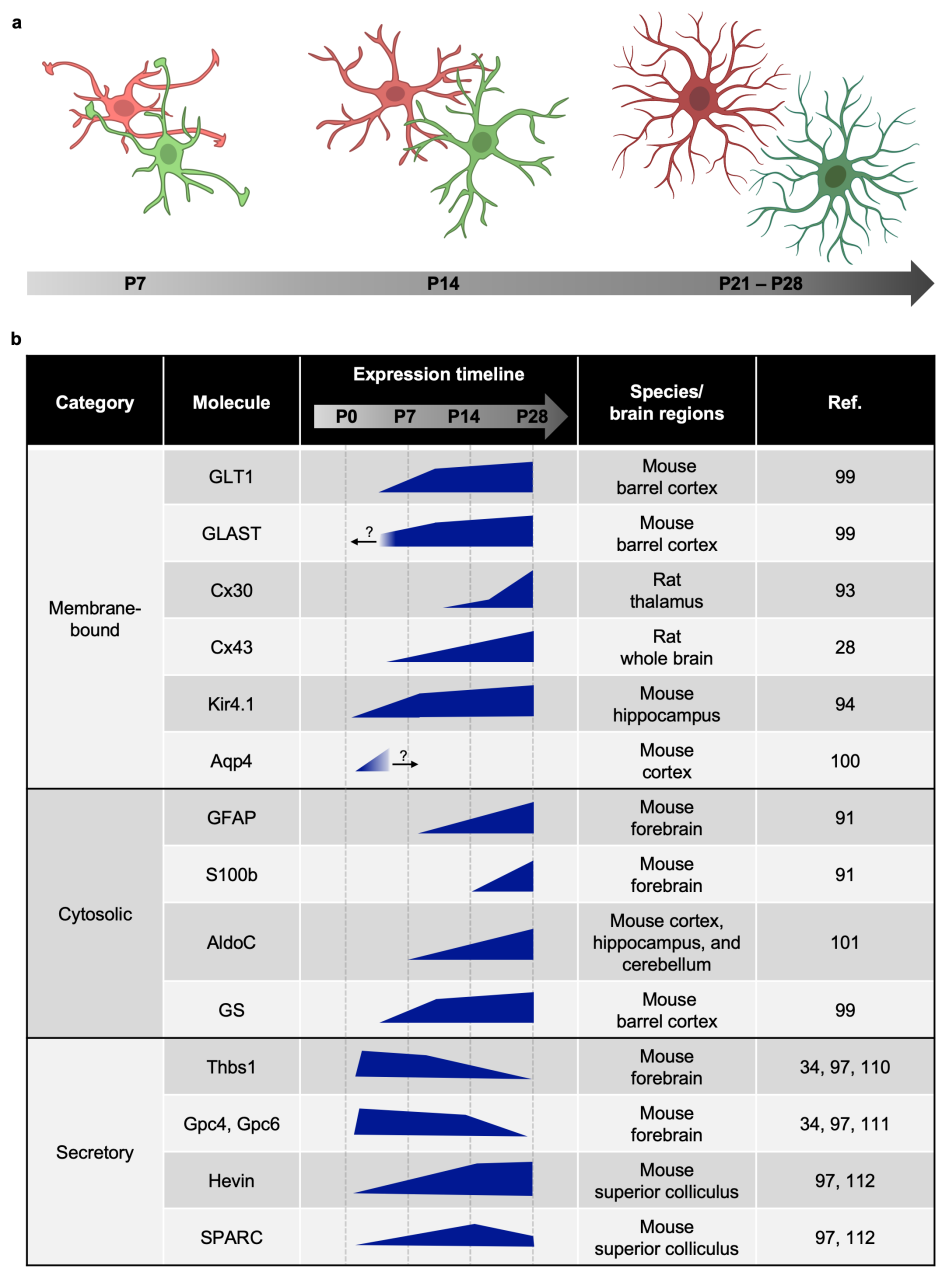
Maturation of astrocytes during postnatal development demonstrated by progressive changes of morphology and gene expression. ( **a**) Morphology of astrocytes matures in a stepwise manner. At postnatal day 7 (P7), astrocytes exhibit few branches and the overall morphology is simple and territories are small. Long protrusions are often seen extending into the territories of the neighboring astrocytes. The boundaries of territories are not well-defined yet. At postnatal day 14 (P14), the number of branches become greater and astrocyte territories become bigger. More branch points grow out of existing branches, resulting in more complex morphology. Long protrusions are seen less, and the boundaries of territories start to emerge. At postnatal day 21 to day 28 (P21–P28), astrocytes develop their characteristic “spongiform” complex morphology, and there is minimal overlap between neighboring astrocytes and clear boundary of each territory. ( **b**) Genes that are induced during astrocyte maturation. Blue polygons indicate expression levels of the genes during development. Question marks indicate that the expression level beyond the developmental stage is unknown.

Early studies in postnatal rats delineated the stages of astrocyte morphogenesis in the hippocampus by using intracellular dye filling in fixed brain slices ^64^. At P7 and P14, astrocytes appear smaller and less ramified, and a dozen long processes stick out from soma and end with filopodia-like structures. The territory of these astrocytes is not well defined, and long extending branches often invade the “territory” of neighboring astrocytes. By P21, most of the filopodia-like structures disappear and fine distal processes appear, resulting in more ramified astrocytes with clear territories and processes with limited overlap among neighboring cells. Concurrently, from P7 to P14, astrocyte morphology becomes more homogeneous, and by P28, spongiform, highly ramified protoplasmic astrocytes are abundant ( Figure 3a).

As astrocyte morphology matures, a specialized structure called PAP also appears at the distal ends of astrocytes. PAPs are extremely fine (<50 nm) distal processes positioned near synapses ^65, 66^. This arrangement allows communication between astrocytes and synapses and originally formed the basis of the concept of the “tripartite synapse” ^64, 67, 68^. To facilitate crosstalk with the synapse, PAPs exhibit high surface-to-volume ratios, providing ample space for the expression of channels, transporters, and receptors in the membranes, including glutamate transporters GLAST and GLT1 ^69^, potassium channel Kir4.1 ^70^, and glutamate receptors mGluR 3 and 5 ^71^ (see the “Molecular maturation” section). With this specialized anatomical arrangement and protein localization, PAPs maintain homeostasis of the local environment at the synaptic cleft ^69, 70^ and also actively crosstalk with synapses and regulate their function ^68, 71^.

Given the close relationship between astrocyte processes and synapses, it is not surprising that neurons or neuronal activity or both participate in astrocyte morphogenesis and PAP plasticity ^41, 72– 82^. Previous studies demonstrated that astrocytes exhibit reduced territory and neuropil infiltration in dark-reared animals, providing evidence for the involvement of neuronal activity in astrocyte morphogenesis ^73, 74^. Several subsequent studies demonstrated that the neurotransmitter glutamate is one molecular mechanism underlying this activity-dependent morphogenesis ^75– 77^. For example, using a VGluT1 KO mouse model in which VGluT1 ^+^ synaptic activity is silenced, Morel *et al*. found that excitatory synaptic activity is required for the growth of astrocyte territory and for perisynaptic astrocyte processes to ensheathe synapses ^75^. Mechanistically, this crosstalk is mediated by an astrocytic glutamate receptor mGluR5-dependent signaling pathway and downstream intracellular Ca ^2+^ activity ^75^. The above findings suggest that the local environment guides astrocyte maturation, resulting in astrocyte processes that facilitate neighboring synapses.

Interestingly, a similar mechanism influences the structural plasticity of PAPs in the adult brain. Using time-lapse imaging on organotypic brain slices and anesthetized adult mouse, Bernardinelli *et al*. found that PAPs are highly motile at a time scale of several minutes ^76^. This structural plasticity is regulated by synaptic activity and is dependent on astrocyte metabotropic glutamate receptor mGluR1/5. Moreover, astrocytic Ca ^2+^ signaling, presumably downstream of mGluR1/5, is both necessary and sufficient for PAP motility. In that study, changes in PAP motility enhanced astrocytic coverage of the synapse and spine stability ^76^. Currently, whether glutamate also regulates PAP maturation and motility during development remains unknown. However, the conserved function of glutamate in controlling astrocyte morphology during development and PAP plasticity in adulthood suggests that glutamate might also play a role in PAP development during the postnatal period.

Neurons also regulate astrocyte morphogenesis and PAP plasticity via contact-dependent mechanisms, including neuroligin–neurexin ^73^, Notch signaling ^72^, and EphA4/ephrine-A3 ^79, 80, 83^. In the case of neuroligin–neurexin interaction, direct contact between astrocytic neuroligins and presynaptic neurexin is required and sufficient for astrocyte morphogenesis and PAP development *in vitro* and *in vivo* ^73^. Moreover, the morphological effect of astrocytic neuroligins is associated with the development of local excitatory synapses. In the case of EphA4/ephrine-A3, neuronal EphA4 and astrocytic ephrin-A3 interact to maintain the proper structure of spine morphology and also are required for proper expression of glutamate transporters in the astrocytic PAPs that surround the spine ^79, 80, 83^. Together, the above studies reveal a contact-dependent reciprocal mechanism between astrocytic synapses and PAPs and demonstrate that astrocyte morphogenesis and synaptic formation and function are tightly linked components that coordinate during the critical period of postnatal synaptogenesis.

Although the biological consequences of neuron–astrocyte interactions are well characterized, the downstream mechanisms by which their contact results in morphological changes or PAP plasticity are still limited. Interestingly, there is a plethora of evidence demonstrating the involvement of actin filaments and their regulators in astrocyte morphological changes, including Arp2/3, N-WASP, small GTPase Rho and Rac, and the effector ROCK, though mostly *in vitro* ^84– 87^. Henceforth, testing whether similar mechanisms exist under physiological conditions *in vivo* upon neuron–astrocyte contact and mediate astrocyte morphological maturation during development would be necessary.

### Molecular maturation

As astrocytes mature, their transcriptional profiles change dramatically to exhibit stage-specific signatures ^14, 19, 21, 34, 88, 89^. The genes that are induced during astrocyte maturation can be categorized into three groups on the basis of their locations: (1) membrane-bound proteins, including GLT1, Cx43, Cx30, Kir4.1, and Aqp4; (2) cytosolic proteins, including GFAP, S100b, AldoC, and GS; and (3) secretory proteins, including Thbs1, Gpc4, Gpc6, Hevin, and SPARC ^28, 42, 90– 101^ ( Figure 3b).

The membrane-bound proteins necessary for astrocytic maturation include channels and receptors, and this group of genes is enriched in subcellular structures such as PAPs and end-feet. Expression of these genes offers astrocytes their characteristic functions during postnatal development. For example, the glutamate transporter GLT1 mediates glutamate uptake and regulates glutamate availability in the synaptic cleft, thus regulating synaptic transmission ^102, 103^. Cx43 and Cx30 form gap junctions to connect neighboring cells, allowing the exchange of molecules (water, glucose, metabolites, and neurotransmitters) and ions (Ca ^2+^, K ^+^, and Na ^+^) over long distances ^104, 105^. Kir4.1 is the major potassium channel of astrocytes and plays an essential role in buffering extracellular potassium built up during action potential. Aqp4 is the most abundant water channel in the brain and is important for water balance and also contributes to synaptic plasticity and learning/memory in the CNS ^27, 106^. During the morphological maturation of astrocytes, neurons often induce these genes ^72, 75, 107– 109^.

The second group of genes is composed of enzymes and cytoskeleton proteins that serve as markers of mature astrocytes. However, each one of these genes labels only a portion of astrocytes, and there is some overlap in expression with other cell types. Comprehensive characterization and immunohistochemical validation of the timeline and astrocyte specificity of these markers during development remain open avenues of investigation. Furthermore, whether and how cytosolic proteins serve as members of combinatorial codes in different brain regions are interesting areas for future research. (Also, see the “Astrocyte differentiation” section.)

Astrocytic neuroactive molecules that regulate synaptogenesis make up the genetic signature of the third and final stage of astrocyte maturation. These molecules include Thbs1, Gpc4, Gpc6, Hevin, and SPARC, each of which regulates a different phase of synaptogenesis. These genes are concurrently expressed in astrocytes at corresponding developmental stages. For example, Thbs1, which is required for the formation of silent synapses, and Gpc4/6, which is required for inducing postsynaptic active synapses, have the highest expression between P0 and P14 in developing astrocytes and are down-regulated thereafter. On the other hand, SPARC, an inhibitor of hevin and a negative regulator of synaptogenesis, is elevated only around P14 and remains expressed thereafter ^34, 110– 112^. The temporal control of these synaptogenic genes suggests a well-defined mechanism that regulates different phases of synaptogenesis.

Despite the above findings, most studies to date have focused on neuron–astrocyte crosstalk via secretory molecules and membrane proteins (that is, glutamate, BDNF, mGluR, Eph–Ephrine, neuroligin–neurexin), and very little investigation of the upstream transcriptional mechanisms governs their expression. Thus, how transcription programs reciprocally respond to neuronal signaling is an important area of future research.

Borrowing a concept from neurons, astrocytes may respond to external environmental cues and tailor their course of maturation to the local environment via transcriptional regulation. With more advanced technology, including single-cell sequencing, super-resolution microscopy, *in vivo* live imaging, membrane-bound optical sensors for neurotransmitters and ions, fluorescence resonance energy transfer techniques, and three-dimensional culture systems that reproduce astrocyte morphology *in vivo*, we should be able to increase our understanding of the mechanisms that mediate crosstalk between neurons and astrocytes, and that facilitate maturation of astrocytes into the right subtype, at the right time and place.

In the future, it will be interesting to answer the following questions: (1) Do any cell types other than neurons (for example, neighboring astrocytes, endothelial cells, oligodendrocytes, or oligodendrocyte precursors) contribute to astrocyte maturation? (2) How does transient Ca ^2+^, an indicator of mature astrocyte activity, develop over this postnatal period? (3) Does Ca ^2+^ activity also mediate astrocyte–neuron crosstalk to facilitate the co-maturation of PAPs and synapses? When addressing these questions, we should bear in mind that astrocytes are very responsive cells that preserve certain progenitor properties even in their mature form, a characteristic supported by recent studies looking into the transformation of reactive astrocytes in response to injury and trans-differentiation into neurons *in vivo* ^113, 114^. Thus, the astrocytic developmental program may be defined by proliferation, differentiation, and maturation over time, and their final refinement is regulated by the local environment, which gives rise to the optimal number, function, and morphology of astrocytes.

## Closing remarks

Since Virchow first discovered astrocytes more than a hundred years ago, much progress has been made in our understanding of the processes by which they are specified, migrate, proliferate, and mature. However, there remain significant gaps in our characterization of these processes and our knowledge regarding molecular mechanisms underlying astrocyte phenotypes. Some crucial outstanding questions include (1) which upstream signaling pathways regulate the proliferation of astrocytes in the postnatal cortex? (2) Do transcription factors serve as immediate early genes that govern astrocyte maturation in accordance with the local synaptic environment? (3) Is Ca ^2+^ activity mature during the period of astrocytic morphological and molecular maturation? With the recent explosion of knowledge and tools in the glial field, the future is bright, and we are looking forward to a better understanding of astrocyte development and the mechanisms underlying it.

## References

[1] John Lin C-C Yu K Hatcher A: Identification of diverse astrocyte populations and their malignant analogs. *Nat Neurosci.* 2017;20(3):396–405. 10.1038/nn.4493 28166219 PMC5824716

[2] Chai H Diaz-Castro B Shigetomi E: Neural Circuit-Specialized Astrocytes: Transcriptomic, Proteomic, Morphological, and Functional Evidence. *Neuron.* 2017;95(3):531–549.e9. 10.1016/j.neuron.2017.06.029 28712653 PMC5811312

[3] Morel L Chiang MSR Higashimori H: Molecular and Functional Properties of Regional Astrocytes in the Adult Brain. *J Neurosci.* 2017;37(36):8706–17. 10.1523/JNEUROSCI.3956-16.2017 28821665 PMC5588463

[4] Herculano-Houzel S: The glia/neuron ratio: How it varies uniformly across brain structures and species and what that means for brain physiology and evolution. *Glia.* 2014;62(9):1377–91. 10.1002/glia.22683 24807023

[5] von Bartheld CS Bahney J Herculano-Houzel S: The search for true numbers of neurons and glial cells in the human brain: A review of 150 years of cell counting. *J Comp Neurol.* 2016;524(18):3865–95. 10.1002/cne.24040 27187682 PMC5063692

[6] Bayraktar OA Fuentealba LC Alvarez-Buylla A: Astrocyte development and heterogeneity. *Cold Spring Harb Perspect Biol.* 2014;7(1):a020362. 10.1101/cshperspect.a020362 25414368 PMC4292163

[7] Glasgow SM Carlson JC Zhu W: Glia-specific enhancers and chromatin structure regulate NFIA expression and glioma tumorigenesis. *Nat Neurosci.* 2017;20(11):1520–8. 10.1038/nn.4638 28892058 PMC5919190

[8] Deneen B Ho R Lukaszewicz A: The transcription factor NFIA controls the onset of gliogenesis in the developing spinal cord. *Neuron.* 2006;52(6):953–68. 10.1016/j.neuron.2006.11.019 17178400

[9] Kang P Lee HK Glasgow SM: Sox9 and NFIA Coordinate a Transcriptional Regulatory Cascade during the Initiation of Gliogenesis. *Neuron.* 2012;74(1):79–94. 10.1016/j.neuron.2012.01.024 22500632 PMC3543821

[10] Nagao M Ogata T Sawada Y: Zbtb20 promotes astrocytogenesis during neocortical development. *Nat Commun.* 2016;7:11102. 10.1038/ncomms11102 27000654 PMC4804180

[11] Bajenaru ML Zhu Y Hedrick NM: Astrocyte-specific inactivation of the neurofibromatosis 1 gene ( *NF1*) is insufficient for astrocytoma formation. *Mol Cell Biol.* 2002;22(14):5100–13. 10.1128/mcb.22.14.5100-5113.2002 12077339 PMC139771

[12] Garcia ADR Doan NB Imura T: GFAP-expressing progenitors are the principal source of constitutive neurogenesis in adult mouse forebrain. *Nat Neurosci.* 2004;7(11):1233–41. 10.1038/nn1340 15494728

[13] Gregorian C Nakashima J Le Belle J: *Pten* deletion in adult neural stem/progenitor cells enhances constitutive neurogenesis. *J Neurosci.* 2009;29(6):1874–86. 10.1523/JNEUROSCI.3095-08.2009 19211894 PMC2754186

[14] Molofsky AV Krenick R Ullian E: Astrocytes and disease: A neurodevelopmental perspective. *Genes Dev.* 2012;26(9):891–907. 10.1101/gad.188326.112 22549954 PMC3347787

[15] Muroyama Y Fujiwara Y Orkin SH: Specification of astrocytes by bHLH protein SCL in a restricted region of the neural tube. *Nature.* 2005;438(7066):360–3. 10.1038/nature04139 16292311

[16] Hochstim C Deneen B Lukaszewicz A: Identification of positionally distinct astrocyte subtypes whose identities are specified by a homeodomain code. *Cell.* 2008;133(3):510–22. 10.1016/j.cell.2008.02.046 18455991 PMC2394859

[17] Tsai HH Li H Fuentealba LC: Regional Astrocyte Allocation Regulates CNS Synaptogenesis and Repair. *Science.* 2012;337(6092):358–62. 10.1126/science.1222381 22745251 PMC4059181

[18] Slezak M Göritz C Niemiec A: Transgenic mice for conditional gene manipulation in astroglial cells. *Glia.* 2007;55(15):1565–76. 10.1002/glia.20570 17823970

[19] Chaboub LS Manalo JM Lee HK: Temporal Profiling of Astrocyte Precursors Reveals Parallel Roles for *Asef* during Development and after Injury. *J Neurosci.* 2016;36(47):11904–17. 10.1523/JNEUROSCI.1658-16.2016 27881777 PMC5125245

[20] Weng Q Wang J Wang J: Single-Cell Transcriptomics Uncovers Glial Progenitor Diversity and Cell Fate Determinants during Development and Gliomagenesis. *Cell Stem Cell.* 2019;24(5):707–723.e8. 10.1016/j.stem.2019.03.006 30982771 PMC6669001

[21] Molofsky AV Glasgow SM Chaboub LS: Expression profiling of Aldh1l1-precursors in the developing spinal cord reveals glial lineage-specific genes and direct Sox9-Nfe2l1 interactions. *Glia.* 2013;61(9):1518–32. 10.1002/glia.22538 23840004 PMC3909648

[22] Zhuo L Theis M Alvarez-Maya I: hGFAP-cre transgenic mice for manipulation of glial and neuronal function in vivo. *Genesis.* 2001;31(2):85–94. 10.1002/gene.10008 11668683

[23] Ghandour MS Langley OK Labourdette G: Specific and artefactual cellular localizations of S 100 protein: An astrocyte marker in rat cerebellum. *Dev Neurosci.* 1981;4(1):66–78. 10.1159/000112742 7011781

[24] Shibata T Yamada K Watanabe M: Glutamate Transporter GLAST Is Expressed in the Radial Glia–Astrocyte Lineage of Developing Mouse Spinal Cord. *J Neurosci.* 1997;17(23):9212–9. 10.1523/JNEUROSCI.17-23-09212.1997 9364068 PMC6573593

[25] Staugaitis SM Zerlin M Hawkes R: Aldolase C/Zebrin II Expression in the Neonatal Rat Forebrain Reveals Cellular Heterogeneity within the Subventricular Zone and Early Astrocyte Differentiation. *J Neurosci.* 2001;21(16):6195–205. 10.1523/JNEUROSCI.21-16-06195.2001 11487642 PMC6763165

[26] Liu Y Wu Y Lee JC: Oligodendrocyte and astrocyte development in rodents: An in situ and immunohistological analysis during embryonic development. *Glia.* 2002;40(1):25–43. 10.1002/glia.10111 12237841

[27] Szu JI Binder DK: The Role of Astrocytic Aquaporin-4 in Synaptic Plasticity and Learning and Memory. *Front Integr Neurosci.* 2016;10:8. 10.3389/fnint.2016.00008 26941623 PMC4764708

[28] Yamamoto T Vukelic J Hertzberg EL: Differential anatomical and cellular patterns of connexin43 expression during postnatal development of rat brain. *Brain Res Dev Brain Res.* 1992;66(2):165–80. 10.1016/0165-3806(92)90077-a 1318799

[29] Furness DN Dehnes Y Akhtar AQ: A quantitative assessment of glutamate uptake into hippocampal synaptic terminals and astrocytes: New insights into a neuronal role for excitatory amino acid transporter 2 (EAAT2). *Neuroscience.* 2008;157(1):80–94. 10.1016/j.neuroscience.2008.08.043 18805467 PMC2775085

[30] Miller SJ Philips T Kim N: Molecularly defined cortical astroglia subpopulation modulates neurons via secretion of Norrin. *Nat Neurosci.* 2019;22(5):741–52. 10.1038/s41593-019-0366-7 30936556 PMC6551209

[31] Morel L Men Y Chiang MSR: Intracortical astrocyte subpopulations defined by astrocyte reporter Mice in the adult brain. *Glia.* 2019;67(1):171–81. 10.1002/glia.23545 30430665

[32] Srinivasan R Lu TY Chai H: New Transgenic Mouse Lines for Selectively Targeting Astrocytes and Studying Calcium Signals in Astrocyte Processes In Situ and In Vivo. *Neuron.* 2016;92(6):1181–95. 10.1016/j.neuron.2016.11.030 27939582 PMC5403514

[33] Sun W Cornwell A Li J: SOX9 Is an Astrocyte-Specific Nuclear Marker in the Adult Brain Outside the Neurogenic Regions. *J Neurosci* 2017;37(17):4493–507. 10.1523/JNEUROSCI.3199-16.2017 28336567 PMC5413187

[34] Cahoy JD Emery B Kaushal A: A transcriptome database for astrocytes, neurons, and oligodendrocytes: a new resource for understanding brain development and function. *J Neurosci.* 2008;28(1):264–78. 10.1523/JNEUROSCI.4178-07.2008 18171944 PMC6671143

[35] Bonni A Sun Y Nadal-Vicens M: Regulation of gliogenesis in the central nervous system by the JAK-STAT signaling pathway. *Science.* 1997;278(5337):477–83. 10.1126/science.278.5337.477 9334309

[36] Barnabé-Heider F Wasylnka JA Fernandes KJL: Evidence that Embryonic Neurons Regulate the Onset of Cortical Gliogenesis via Cardiotrophin-1. *Neuron.* 2005;48(2):253–65. 10.1016/j.neuron.2005.08.037 16242406

[37] Ito K Noguchi A Uosaki Y: *Gfap* and *Osmr* regulation by BRG1 and STAT3 via interchromosomal gene clustering in astrocytes. *Mol Biol Cell.* 2018;29(2):209–19. 10.1091/mbc.E17-05-0271 29142070 PMC5909932

[38] Molofsky AV Kelley KW Tsai HH: Astrocyte-encoded positional cues maintain sensorimotor circuit integrity. *Nature.* 2014;509(7499):189–94. 10.1038/nature13161 24776795 PMC4057936

[39] Kelley KW Ben Haim L Schirmer L: Kir4.1-Dependent Astrocyte-Fast Motor Neuron Interactions Are Required for Peak Strength. *Neuron.* 2018;98(2):306–319.e7. 10.1016/j.neuron.2018.03.010 29606582 PMC5919779

[40] van Dyck LI Morrow EM: Genetic control of postnatal human brain growth. *Curr Opin Neurol.* 2017;30(1):114–24. 10.1097/WCO.0000000000000405 27898583 PMC5340196

[41] Bandeira F Lent R Herculano-Houzel S: Changing numbers of neuronal and non-neuronal cells underlie postnatal brain growth in the rat. *Proc Natl Acad Sci U S A.* 2009;106(33):14108–13. 10.1073/pnas.0804650106 19666520 PMC2729028

[42] Allen NJ Eroglu C: Cell Biology of Astrocyte-Synapse Interactions. *Neuron.* 2017;96(3):697–708. 10.1016/j.neuron.2017.09.056 29096081 PMC5687890

[43] Kriegstein A Alvarez-Buylla A: The Glial Nature of Embryonic and Adult Neural Stem Cells. *Annu Rev Neurosci.* 2009;32:149–84. 10.1146/annurev.neuro.051508.135600 19555289 PMC3086722

[44] Tabata H: Diverse subtypes of astrocytes and their development during corticogenesis. *Front Neurosci.* 2015;9:114. 10.3389/fnins.2015.00114 25904839 PMC4387540

[45] Ge WP Jia JM: Local production of astrocytes in the cerebral cortex. *Neuroscience.* 2016;323:3–9. 10.1016/j.neuroscience.2015.08.057 26343293 PMC4779064

[46] Schmechel DE Rakic P: A golgi study of radial glial cells in developing monkey telencephalon: Morphogenesis and transformation into astrocytes. *Anat Embryol.* 1979;156(2):115–52. 10.1007/bf00300010 111580

[47] Voigt T: Development of glial cells in the cerebral wall of ferrets: Direct tracing of their transformation from radial glia into astrocytes. *J Comp Neurol.* 1989;289(1):74–88. 10.1002/cne.902890106 2808761

[48] deAzevedo LC Fallet C Moura-Neto V: Cortical radial glial cells in human fetuses: Depth-correlated transformation into astrocytes. *J Neurobiol.* 2003;55(3):288–98. 10.1002/neu.10205 12717699

[49] Noctor SC Martínez-Cerdeño V Ivic L: Cortical neurons arise in symmetric and asymmetric division zones and migrate through specific phases. *Nat Neurosci.* 2004;7(2):136–44. 10.1038/nn1172 14703572

[50] Burns KA Murphy B Danzer SC: Developmental and post-injury cortical gliogenesis: A Genetic fate-mapping study with Nestin-CreER mice. *Glia.* 2009;57(10):1115–29. 10.1002/glia.20835 19115384 PMC4286201

[51] Magavi S Friedmann D Banks G: Coincident generation of pyramidal neurons and protoplasmic astrocytes in neocortical columns. *J Neurosci.* 2012;32(14):4762–72. 10.1523/JNEUROSCI.3560-11.2012 22492032 PMC3643505

[52] Levison SW Goldman JE: Both oligodendrocytes and astrocytes develop from progenitors in the subventricular zone of postnatal rat forebrain. *Neuron.* 1993;10(2):201–12. 10.1016/0896-6273(93)90311-e 8439409

[53] Levison SW Chuang C Abramson BJ: The migrational patterns and developmental fates of glial precursors in the rat subventricular zone are temporally regulated. *Development.* 1993;119(3):611–22. 8187632 10.1242/dev.119.3.611

[54] Luskin MB McDermott K: Divergent lineages for oligodendrocytes and astrocytes originating in the neonatal forebrain subventricular zone. *Glia.* 1994;11(3):211–26. 10.1002/glia.440110302 7960027

[55] Hirano M Goldman JE: Gliogenesis in rat spinal cord: Evidence for origin of astrocytes and oligodendrocytes from radial precursors. *J Neurosci Res.* 1988;21(2-4):155–67. 10.1002/jnr.490210208 3216418

[56] Tien AC Tsai HH Molofsky AV: Regulated temporal-spatial astrocyte precursor cell proliferation involves BRAF signalling in mammalian spinal cord. *Development.* 2012;139(14):2477–87. 10.1242/dev.077214 22675209 PMC3383225

[57] Price J Thurlow L: Cell lineage in the rat cerebral cortex: a study using retroviral-mediated gene transfer. *Development.* 1988;104(3):473–82. 3151483 10.1242/dev.104.3.473

[58] Ge WP Miyawaki A Gage FH: Local generation of glia is a major astrocyte source in postnatal cortex. *Nature.* 2012;484(7394):376–80. 10.1038/nature10959 22456708 PMC3777276

[59] Moroni RF Deleo F Regondi MC: Proliferative cells in the rat developing neocortical grey matter: new insights into gliogenesis. *Brain Struct Funct.* 2018;223(9):4053–66. 10.1007/s00429-018-1736-8 30132245

[60] Sohn J Orosco L Guo F: The subventricular zone continues to generate corpus callosum and rostral migratory stream astroglia in normal adult mice. *J Neurosci..* 2015;35(9):3756–63. 10.1523/JNEUROSCI.3454-14.2015 25740506 PMC6605576

[61] García-Marqués J López-Mascaraque L: Clonal Identity Determines Astrocyte Cortical Heterogeneity. *Cereb Cortex.* 2013;23(6):1463–72. 10.1093/cercor/bhs134 22617854

[62] Huang Z Hu J Pan J: YAP Stabilizes SMAD1 and Promotes BMP2-induced Neocortical Astrocytic Differentiation. *Development.* 2016;143(13):2398–409. 10.1242/dev.130658 27381227 PMC4958318

[63] Haim LB Rowitch DH: Functional Diversity of Astrocytes in Neural Circuit Regulation. *Nat Rev Neurosci.* 2017;18(1):31–41. 10.1038/nrn.2016.159 27904142

[64] Bushong EA Martone ME Ellisman MH: Maturation of Astrocyte Morphology and the Establishment of Astrocyte Domains During Postnatal Hippocampal Development. *Int J Dev Neurosci.* 2004;22(2):73–86. 10.1016/j.ijdevneu.2003.12.008 15036382

[65] Octeau JC Chai H Jiang R: An Optical Neuron-Astrocyte Proximity Assay at Synaptic Distance Scales. *Neuron.* 2018;98(1):49–66.e9. 10.1016/j.neuron.2018.03.003 29621490 PMC5916847

[66] Derouiche A Frotscher M: Peripheral Astrocyte Processes: Monitoring by Selective Immunostaining for the Actin-Binding ERM Proteins. *Glia.* 2001;36(3):330–41. 10.1002/glia.1120 11746770

[67] Freeman MR: Specification and Morphogenesis of Astrocytes. *Science.* 2010;330(6005):774–8. 10.1126/science.1190928 21051628 PMC5201129

[68] Savtchouk I Volterra A: Gliotransmission: Beyond Black-and-White. *J Neurosci.* 2018;38(1):14–25. 10.1523/JNEUROSCI.0017-17.2017 29298905 PMC6705815

[69] Chaudhry FA Lehre KP van Lookeren Campagne M: Glutamate transporters in glial plasma membranes: highly differentiated localizations revealed by quantitative ultrastructural immunocytochemistry. *Neuron.* 1995;15(3):711–20. 10.1016/0896-6273(95)90158-2 7546749

[70] Higashi K Fujita A Inanobe A: An Inwardly Rectifying K(+) Channel, Kir4.1, Expressed in Astrocytes Surrounds Synapses and Blood Vessels in Brain. *Am J Physiol Cell Physiol.* 2001;281(3):C922–31. 10.1152/ajpcell.2001.281.3.C922 11502569

[71] Lavialle M Aumann G Anlauf E: Structural plasticity of perisynaptic astrocyte processes involves ezrin and metabotropic glutamate receptors. *Proc Natl Acad Sci U S A.* 2011;108(31):12915–9. 10.1073/pnas.1100957108 21753079 PMC3150955

[72] Hasel P Dando O Jiwaji Z: Neurons and neuronal activity control gene expression in astrocytes to regulate their development and metabolism. *Nat Commun.* 2017;8:15132. 10.1038/ncomms15132 28462931 PMC5418577

[73] Stogsdill JA Ramirez J Liu D: Astrocytic neuroligins control astrocyte morphogenesis and synaptogenesis. *Nature.* 2017;551(7679):192–197. 10.1038/nature24638 29120426 PMC5796651

[74] Müller CM: Dark-rearing retards the maturation of astrocytes in restricted layers of cat visual cortex. *Glia.* 1990;3(6):487–94. 10.1002/glia.440030607 2148551

[75] Morel L Higashimori H Tolman M: VGluT1 ^+^ neuronal glutamatergic signaling regulates postnatal developmental maturation of cortical protoplasmic astroglia. *J Neurosci.* 2014;34(33):10950–62. 10.1523/JNEUROSCI.1167-14.2014 25122895 PMC4131010

[76] Bernardinelli Y Randall J Janett E: Activity-Dependent Structural Plasticity of Perisynaptic Astrocytic Domains Promotes Excitatory Synapse Stability. *Curr Biol.* 2014;24(15):1679–88. 10.1016/j.cub.2014.06.025 25042585

[77] Genoud C Quairiaux C Steiner P: Plasticity of astrocytic coverage and glutamate transporter expression in adult mouse cortex. *PLoS Biol.* 2006;4(11):e343. 10.1371/journal.pbio.0040343 17048987 PMC1609127

[78] Holt LM Hernandez RD Pacheco NL: Astrocyte morphogenesis is dependent on BDNF signaling via astrocytic TrkB.T1. *Elife.* 2019;8: pii: e44667. 10.7554/eLife.44667 31433295 PMC6726422

[79] Murai KK Nguyen LN Irie F: Control of hippocampal dendritic spine morphology through ephrin-A3/EphA4 signaling. *Nat Neurosci.* 2003;6(2):153–60. 10.1038/nn994 12496762

[80] Filosa A Paixão S Honsek SD: Neuron-glia communication via EphA4/ephrin-A3 modulates LTP through glial glutamate transport. *Nat Neurosci.* 2009;12(10):1285–92. 10.1038/nn.2394 19734893 PMC3922060

[81] Garrett AM Weiner JA: Control of CNS synapse development by {gamma}-protocadherin-mediated astrocyte-neuron contact. *J Neurosci.* 2009;29(38):11723–31. 10.1523/JNEUROSCI.2818-09.2009 19776259 PMC2778296

[82] Medvedev N Popov V Henneberger C: Glia selectively approach synapses on thin dendritic spines. *Philos Trans R Soc Lond B Biol Sci.* 2014;369(1654):20140047. 10.1098/rstb.2014.0047 25225105 PMC4173297

[83] Carmona MA Murai KK Wang L: Glial ephrin-A3 regulates hippocampal dendritic spine morphology and glutamate transport. *Proc Natl Acad Sci U S A.* 2009;106(30):12524–9. 10.1073/pnas.0903328106 19592509 PMC2718351

[84] Racchetti G D'Alessandro R Meldolesi J: Astrocyte stellation, a process dependent on Rac1 is sustained by the regulated exocytosis of enlargeosomes. *Glia.* 2012;60(3):465–75. 10.1002/glia.22280 22144092 PMC3306795

[85] Murk K Blanco Suarez EM Cockbill LM: The antagonistic modulation of Arp2/3 activity by N-WASP, WAVE2 and PICK1 defines dynamic changes in astrocyte morphology. *J Cell Sci.* 2013;126(Pt 17):3873–83. 10.1242/jcs.125146 23843614 PMC3757329

[86] John GR Chen L Rivieccio MA: Interleukin-1beta induces a reactive astroglial phenotype via deactivation of the Rho GTPase-Rock axis. *J Neurosci.* 2004;24(11):2837–45. 10.1523/JNEUROSCI.4789-03.2004 15028778 PMC6729504

[87] Nishida H Okabe S: Direct astrocytic contacts regulate local maturation of dendritic spines. *J Neurosci.* 2007;27(2):331–40. 10.1523/JNEUROSCI.4466-06.2007 17215394 PMC6672072

[88] Li J Khankan RR Caneda C: Astrocyte-to-astrocyte contact and a positive feedback loop of growth factor signaling regulate astrocyte maturation. *Glia.* 2019;67(8):1571–1597. 10.1002/glia.23630 31033049 PMC6557696

[89] Zhang Y Sloan SA Clarke LE: Purification and Characterization of Progenitor and Mature Human Astrocytes Reveals Transcriptional and Functional Differences with Mouse. *Neuron.* 2016;89(1):37–53. 10.1016/j.neuron.2015.11.013 26687838 PMC4707064

[90] Baba H Nakahira K Morita N: GFAP gene expression during development of astrocyte. *Dev Neurosci.* 1997;19(1):49–57. 10.1159/000111185 9078433

[91] Raponi E Agenes F Delphin C: S100B expression defines a state in which GFAP-expressing cells lose their neural stem cell potential and acquire a more mature developmental stage. *Glia.* 2007;55(2):165–77. 10.1002/glia.20445 17078026 PMC2739421

[92] Furuta A Rothstein JD Martin LJ: Glutamate Transporter Protein Subtypes Are Expressed Differentially during Rat CNS Development. *J Neurosci.* 1997;17(21):8363–75. 10.1523/JNEUROSCI.17-21-08363.1997 9334410 PMC6573756

[93] Nagy JI Patel D Ochalski PAY: Connexin30 in rodent, cat and human brain: Selective expression in gray matter astrocytes, co-localization with connexin43 at gap junctions and late developmental appearance. *Neuroscience.* 1999;88(2):447–68. 10.1016/s0306-4522(98)00191-2 10197766

[94] Seifert G Hüttmann K Binder DK: Analysis of astroglial K+ channel expression in the developing hippocampus reveals a predominant role of the Kir4.1 subunit. *J Neurosci.* 2009;29(23):7474–88. 10.1523/JNEUROSCI.3790-08.2009 19515915 PMC6665420

[95] Higashimori H Sontheimer H: Role of Kir4.1 channels in growth control of glia. *Glia.* 2007;55(16):1668–79. 10.1002/glia.20574 17876807 PMC2040118

[96] Engel M Do-Ha D Muñoz SS: Common pitfalls of stem cell differentiation: A guide to improving protocols for neurodegenerative disease models and research. *Cell Mol Life Sci.* 2016;73(19):3693–709. 10.1007/s00018-016-2265-3 27154043 PMC5002043

[97] Farhy-Tselnicker I Allen NJ: Astrocytes, neurons, synapses: A tripartite view on cortical circuit development. *Neural Dev.* 2018;13(1):7. 10.1186/s13064-018-0104-y 29712572 PMC5928581

[98] Nwaobi SE Lin E Peramsetty SR: DNA methylation functions as a critical regulator of Kir4.1 expression during CNS development. *Glia.* 2014;62(3):411–27. 10.1002/glia.22613 24415225 PMC3991476

[99] Voutsinos-Porche B Knott G Tanaka K: Glial Glutamate Transporters and Maturation of the Mouse Somatosensory Cortex. *Cereb Cortex.* 2003;13(10):1110–21. 10.1093/cercor/13.10.1110 12967927

[100] Fallier-Becker P Vollmer JP Bauer H-C: Onset of aquaporin-4 expression in the developing mouse brain. *Int J Dev Neurosci.* 2014;36:81–9. 10.1016/j.ijdevneu.2014.06.001 24915007

[101] Walther EU Dichgans M Maricich SM: Genomic sequences of aldolase C (Zebrin II) direct *lacZ* expression exclusively in non-neuronal cells of transgenic mice. *Proc Natl Acad Sci U S A.* 1998;95(5):2615–20. 10.1073/pnas.95.5.2615 9482935 PMC19434

[102] Rose CR Felix L Zeug A: Astroglial Glutamate Signaling and Uptake in the Hippocampus. *Front Mol Neurosci.* 2018;10:41. 10.3389/fnmol.2017.00451 29386994 PMC5776105

[103] Pannasch U Freche D Dallérac G: Connexin 30 sets synaptic strength by controlling astroglial synapse invasion. *Nat Neurosci.* 2014;17(4):549–58. 10.1038/nn.3662 24584052

[104] Lanciotti A Brignone M Bertini E: Astrocytes: Emerging stars in leukodystrophy pathogenesis. *Transl Neurosci.* 2013;4(2):356. 10.2478/s13380-013-0118-1 24340223 PMC3856885

[105] Mayorquin C Rodriguez AV Sutachan JJ: Connexin-Mediated Functional and Metabolic Coupling Between Astrocytes and Neurons. *Front Mol Neurosci.* 2018;11:118. 10.3389/fnmol.2018.00118 29695954 PMC5905222

[106] Woo J Kim JE Im JJ: Astrocytic water channel aquaporin-4 modulates brain plasticity in both mice and humans: a potential gliogenetic mechanism underlying language-associated learning. *Mol Psychiatry.* 2018;23(4):1021–30. 10.1038/mp.2017.113 29565042

[107] Yang Y Gozen O Watkins A: Presynaptic regulation of astroglial excitatory neurotransmitter transporter GLT1. *Neuron.* 2009;61(6):880–94. 10.1016/j.neuron.2009.02.010 19323997 PMC2743171

[108] Swanson RA Liu J Miller JW: Neuronal Regulation of Glutamate Transporter Subtype Expression in Astrocytes. *J Neurosci.* 1997;17(3):932–40. 10.1523/JNEUROSCI.17-03-00932.1997 8994048 PMC6573161

[109] Koulakoff A Ezan P Giaume C: Neurons control the expression of connexin 30 and connexin 43 in mouse cortical astrocytes. *Glia.* 2008;56(12):1299–311. 10.1002/glia.20698 18512249

[110] Christopherson KS Ullian EM Stokes CCA: Thrombospondins Are Astrocyte-Secreted Proteins that Promote CNS Synaptogenesis. *Cell.* 2005;120(3):421–33. 10.1016/j.cell.2004.12.020 15707899

[111] Allen NJ Bennett ML Foo LC: Astrocyte glypicans 4 and 6 promote formation of excitatory synapses via GluA1 AMPA receptors. *Nature.* 2012;486(7403):410–4. 10.1038/nature11059 22722203 PMC3383085

[112] Kucukdereli H Allen NJ Lee AT: Control of excitatory CNS synaptogenesis by astrocyte-secreted proteins Hevin and SPARC. *Proc Natl Acad Sci U S A.* 2011;108(32):E440–9. 10.1073/pnas.1104977108 21788491 PMC3156217

[113] Liu Y Miao Q Yuan J: *Ascl1* Converts Dorsal Midbrain Astrocytes into Functional Neurons *In Vivo*. *J Neurosci.* 2015;35(25):9336–55. 10.1523/JNEUROSCI.3975-14.2015 26109658 PMC6605193

[114] Sofroniew MV: Astrogliosis. *Cold Spring Harb Perspect Biol.* 2015;7(2):a020420. 10.1101/cshperspect.a020420 25380660 PMC4315924

